# Carfentrazone-ethyl resistance in an *Amaranthus tuberculatus* population is not mediated by amino acid alterations in the PPO2 protein

**DOI:** 10.1371/journal.pone.0215431

**Published:** 2019-04-15

**Authors:** Olivia A. Obenland, Rong Ma, Sarah R. O’Brien, Anatoli V. Lygin, Dean E. Riechers

**Affiliations:** Department of Crop Sciences, University of Illinois, Urbana, IL, United States of America; Texas A&M University, UNITED STATES

## Abstract

To date, the only known mechanism conferring protoporphyrinogen IX oxidase (PPO)-inhibitor resistance in waterhemp (*Amaranthus tuberculatus*) is a glycine deletion in PPO2 (ΔG210), which results in cross-resistance to foliar PPO-inhibiting herbicides. However, a metabolism-based, HPPD-inhibitor resistant waterhemp population from Illinois (named SIR) was suspected of having a non-target site resistance (NTSR) mechanism due to its resistance to carfentrazone-ethyl (CE) but sensitivity to diphenylethers (DPEs). In greenhouse experiments, SIR sustained less injury than two PPO inhibitor-sensitive populations (WCS and SEN) after applying a field-use rate of CE, and after initial rapid necrosis, regrowth of SIR plants was comparable to a known PPO inhibitor-resistant population (ACR) possessing the ΔG210 mutation. Dose-response analysis determined 50% growth reduction rates in CE-resistant (SIR and ACR) and sensitive (SEN) waterhemp populations, which showed SIR was 30-fold resistant compared to SEN and two-fold more resistant than ACR. Deduced amino acid sequences derived from SIR *PPX2* partial cDNAs did not contain the ΔG210 mutation found in ACR or other target-site mutations that confer PPO-inhibitor resistance previously reported in Palmer amaranth (*Amaranthus palmeri*). Although several SIR cDNAs contained amino acid substitutions, none were uniform among samples. Additionally, SIR plants treated with malathion and CE showed a significant reduction in biomass accumulation compared to CE alone. These results indicate robust CE resistance in SIR is not mediated by amino acid changes in the PPO2 protein, but instead resistance may be conferred through a NTSR mechanism such as enhanced herbicide metabolism.

## Introduction

Protoporphyrinogen IX oxidase (PPO) is an essential enzyme in the biosynthesis of chlorophyll and heme in plants [1]. When PPO is inhibited by herbicides, protoporphyrinogen IX leaks into the cytoplasm, which oxidizes to form highly photodynamic protoporphyrin IX [1]. In the cytosol, protoporphyrin IX generates singlet oxygen, a reactive oxygen species that causes rapid lipid peroxidation [1]. Affected plants display symptoms including wilting, chlorosis, bleaching, desiccation, and necrosis within hours after treatment, and death can occur in a few days [2].

Diphenylethers (DPEs), aryl-triazinones, *N*-phenylphthalimides, and pyrimidinediones are common classes of PPO-inhibiting herbicides applied to agronomic crops primarily for broadleaf weed control [2], with thirteen PPO inhibitor-resistant weed species reported worldwide [3]. Economically important crops, such as maize (*Zea mays)* and soybean (*Glycine max*), possess natural tolerance to these herbicides [1]. While the popularity of transgenic glyphosate-resistant crops contributed to the decline of PPO-inhibiting herbicide usage, there has been a resurgence of interest in these herbicides for controlling glyphosate-resistant weeds [4].

Resistance to PPO-inhibiting herbicides in a field population of waterhemp (*Amaranthus tuberculatus*) was first discovered in 2001 [4, 5]. Mechanistic research later determined that PPO-inhibitor resistance is conferred by a trinucleotide deletion in the *PPX2* gene (ΔG210) which encodes both plastid and mitochondrial isoforms of the PPO2 enzyme through a dual-targeting mechanism [4, 6]. Currently, the ΔG210 mutation is the only known mechanism that confers PPO-inhibitor resistance in waterhemp, and this mutation also has been detected in a close relative, Palmer amaranth (*Amaranthus palmeri*) [4, 5, 7]. A substitution of arginine-128 to either glycine or methionine (R128G/M) in PPO2 confers resistance to PPO inhibitors in Palmer amaranth, which is homologous to the arginine-98 site of PPO2 in common ragweed (*Ambrosia artemisiifolia*) where a substitution with leucine confers PPO-inhibitor resistance [8]. Additional amino acid substitutions that confer broad-spectrum resistance to PPO inhibitors have been reported in Palmer amaranth, such as glycine to alanine (G399A), glycine to glutamic acid (G114E), and serine to isoleucine (S149I) [9]. However, there is also evidence of a non-target site resistance (NTSR) mechanism to the postemergence DPE fomesafen in a Palmer amaranth population from Arkansas [9]. Given the dioecious, outcrossing nature and resulting genetic diversity of waterhemp and Palmer amaranth [10], it is not surprising for these species to develop multiple target-site mutations and diverse mechanisms that confer resistance to the same family of herbicides since any mutation that arises in an individual can be transferred within a population and to other populations. In the future, target-site mutations conferring resistance to PPO inhibitors discovered in Palmer amaranth may be discovered in waterhemp, especially the arginine-128 substitution to glycine since only one nucleotide change would be required in the waterhemp *PPX2* gene [8].

Waterhemp populations from Stanford, Illinois (SIR) and Nebraska (NEB) possess metabolism-based, 4-hydroxyphenylpyruvate-dioxygenase (HPPD)-inhibitor resistance, likely due to enhanced cytochrome P450 monooxygenase (P450) activity [11, 12]. While the SIR population is sensitive to foliar DPEs [13], we hypothesized that SIR and NEB may have a rapid detoxification-mediated mechanism to other classes of PPO inhibitors [14–16] or a novel target-site mutation that confers resistance to certain PPO-inhibiting herbicides. The objectives of this research were to (1) determine if two metabolism-based, HPPD inhibitor-resistant waterhemp populations (NEB and SIR) exhibit non-uniform resistance to several classes of PPO inhibitors, (2) perform a dose-response study to quantify levels of resistance to PPO-inhibiting herbicide(s) to which these waterhemp populations displayed resistance in Objective 1, (3) sequence and compare several *PPX2* partial cDNAs from PPO inhibitor-resistant population(s) to identify any sequence polymorphisms present, and (4) determine if the P450-inhibitor, malathion, applied with PPO-inhibiting herbicide(s) increased herbicidal activity.

## Materials and methods

### Plant materials

Two metabolism-based, HPPD inhibitor-resistant waterhemp populations, NEB and SIR, were compared to PPO inhibitor-resistant and -sensitive waterhemp populations. Mesotrione resistance in NEB is mediated by rapid P450-catalyzed hydroxylation of the parent compound [12]. The SIR population was sampled from the same field as a metabolism-based (via P450), HPPD inhibitor-resistant population (named MCR for McLean County, Illinois Resistant) [11] that previously displayed sensitivity to DPEs in both field and greenhouse conditions [13]. The PPO inhibitor-resistant population from Adams County, Illinois (ACR) is uniformly lactofen-resistant and possesses the ΔG210 codon deletion, and an herbicide-sensitive population from Wayne County, Illinois (WCS) does not have this codon deletion [6]. Another sensitive population (SEN) used in previous research was included for additional comparison [12].

### Response of waterhemp populations to PPO-inhibiting herbicides in the greenhouse

At least one herbicide from each class of PPO inhibitors was selected for the experiment: fomesafen (Flexstar 1.88 EC; Syngenta, Greensboro, NC), oxyfluorfen (Goal 2XL; Dow Agrosciences LLC, Indianapolis, IN), carfentrazone-ethyl (CE) (Aim 2.0 EC; FMC Corp., Philadelphia, PA), saflufenacil (Sharpen; BASF Corp., Research Triangle Park, NC), and flumiclorac-pentyl (Resource; Valent USA Corp., Walnut Creek, CA). Fomesafen and oxyfluorfen are both DPEs; however the relatively slowly metabolized oxyfluorfen served as a comparison to fomesafen, which is rapidly metabolized in soybean through glutathione *S*-transferase-mediated cleavage of the diphenylether bond [2]. The PPO-inhibiting herbicides CE, saflufenacil, and flumiclorac-pentyl belong to aryl-triazinone, pyrimidinedione, and *N*-phenylphthalimide classes, respectively [2]. Fomesafen, CE, and saflufenacil are labeled for waterhemp control, while oxyfluorfen and flumiclorac-pentyl are labeled for control of other *Amaranthus* species. A previous study demonstrated that ACR is 2.9-fold resistant to flumiclorac-pentyl relative to WCS [17]. Application rates for oxyfluorfen and flumiclorac-pentyl were based on control or suppression rates of related *Amaranthus* species because neither herbicide is labeled for use on waterhemp.

Plants from each population were grown using previously described methods for waterhemp [11, 18]. Seeds were planted in 12 x 12 cm trays containing a commercial potting medium (Sun Gro Horticulture, Bellevue, WA) and placed in a growth chamber with conditions of 28/22°C day/night with a 16:8 h photoperiod. When seedlings reached 2 cm they were transplanted into 80 cm^3^ pots, and at 4 cm each seedling was transplanted into 950 cm^3^ pots containing a 3:1:1:1 mixture of potting mix:soil:peat:sand with slow-release fertilizer (Everris Osmocote Classic 13-13-13; BFG Supply, Burton, OH). Seedlings were moved to a greenhouse with the same temperature and photoperiod conditions. Natural light was supplemented with halide lamps delivering 800 μmol m^−2^ s^−1^ photon flux to the plant canopy.

Plants were treated at a height of 9–11 cm using a compressed air research sprayer calibrated to deliver 187 L ha^-1^ at 275 kPa with an even flat-fan nozzle. Five plants (each plant represents one replication) from each population were treated with field-use rates, including: 25 g ai ha^-1^ saflufenacil plus 1% (v/v) methylated seed oil (MSO), 560 g ai ha^-1^ oxyfluorfen plus 0.25% (v/v) nonionic surfactant (NIS), 350 g ai ha^-1^ fomesafen plus 1% (v/v) MSO, 60 g ai ha^-1^ flumiclorac-pentyl plus 1.25% (v/v) MSO, or 14 g ai ha^-1^ CE plus 1% (v/v) crop oil concentrate (COC). In addition to these spray adjuvants, 2.5% (v/v) liquid ammonium sulfate (AMS) was included with each herbicide treatment. Five control plants from each population were treated with only the adjuvant combination used for flumiclorac-pentyl (1.25% (v/v) MSO and 2.5% (v/v) AMS). After spraying, plants were returned to the greenhouse and arranged in a completely randomized design for the duration of the experiment. At 10 days after treatment (DAT), plants were cut at the soil line, bagged, and placed in an oven at 65°C for drying. After 7 days of drying, weights were recorded and compared to their respective controls. The experiment was performed twice independently and data were pooled for further statistical analysis as described below.

### Carfentrazone-ethyl dose-response analysis

Based on the responses of each waterhemp population to PPO-inhibiting herbicides in the greenhouse, ACR, SIR, and SEN were selected to perform a CE dose-response study. Plants were propagated and treated with CE using the same methods described in the previous greenhouse experiment. Three plants (each plant representing one replication) from each population were treated with seven rates of CE using the same adjuvants previously mentioned, and three control plants from each population were treated with adjuvants only. Rates for CE were based on the field-use rate of 14 g ai ha^-1^, which is labeled for controlling waterhemp plants approximately 10 cm in height. The CE rates for SIR, SEN, and ACR were 0.44, 1.4, 4.4, 14, 44, 140, and 440 g ai ha^-1^; 0.04, 0.14, 0.44, 1.4, 4.4, 14, and 44 g ai ha^-1^; and 0.44, 1.4, 4.4, 14, 140, 440, and 900 g ai ha^-1^, respectively. The highest CE rate (900 g ai ha^-1^) was only included for ACR plants due to our initial hypothesis that the ΔG210 mutation would confer the highest level of resistance among populations. Plants were arranged in a completely randomized design under the same greenhouse conditions, and 10 DAT plants were collected and dried using the same methods previously mentioned. This experiment was performed independently three times, and the combined dry weights of all plants were compared to their respective control plants to determine a 50% growth reduction rate of CE (GR_50_) using methods previously described by Hausman *et al*. [18].

### Sequence comparisons of *PPX2* in waterhemp populations

Approximately 0.5 g of tissue was collected from ACR, SIR, and SEN plants by harvesting the youngest leaves from nontreated plants. Total RNA extraction was performed using methods described by Evans *et al*. [19]. The RNA concentrations were determined with a NanoDrop spectrometer, and RNA quality was determined visually by formaldehyde-agarose gel electrophoresis. First-strand cDNA synthesis was performed using the Maxima H-Minus cDNA Synthesis kit with Master Mix (Thermo-Fisher Scientific, Waltham, MA). Polymerase chain reaction (PCR) primers (Table 1) were designed to generate a nearly full-length cDNA (amplicon length was approximately 1580-bp) in order to detect both the ΔG210 codon deletion and R128 substitutions previously found in Palmer amaranth [8]. Reverse transcription (RT)-PCR was performed with recombinant *Taq* polymerase (New England BioLabs, Ipswich, MA; 5 U uL^-1^) using the following parameters: initial denaturing at 95°C for 3 min, followed by 38 amplification cycles of 94°C for 50 s, 58°C for 40 s, and 72°C for 75 s, and final extension at 72°C for 8 min. The RT-PCR products were visualized with a 1.5% agarose gel stained with ethidium bromide and their lengths were verified as ~1.6 kb. Primers and dNTPs were removed from PCR reactions using the QIAquick PCR Purification Kit (Qiagen, Valencia, CA).

**Table 1:** Sequences of primers and internal primers designed for RT-PCR amplification and sequencing of *PPX2* cDNAs from waterhemp.

Name	Purpose	Sequence (5’-3’)
PCR Forward	PCR and Sequencing	CACCTTTCACCAAACCTTGC
PCR Reverse	PCR and Sequencing	GCGGTCTTCTCATCCATCTT
M13F ^a^	Sequencing	GTAAAACGACGGCCAG
M13R	Sequencing	CAGGAAACAGCTATGAC
Internal Primer 1^b^	Sequencing	ATCCCGCTGCACTACTCAC
Internal Primer 2	Sequencing	GTGAGGTGCTGTCCTTGTCA
Internal Primer 3	Sequencing	GTCACTGCTCCAATTCGCA
Internal Primer 4	Sequencing	CTCTTTTGGAGCAACGCATT

^a^ M13F and M13R are plasmid primers provided in the TOPO TA Cloning Kit (Invitrogen, Waltham, MA).

^b^ The reverse complements of the internal primers were also used to sequence the antisense strand of *PPX2* cDNAs as necessary.

After ligating the purified amplicons into the pCR 4-TOPO cloning vector (TOPO TA Cloning Kit, Invitrogen, Waltham, MA), plasmids were transformed into competent *E*. *coli* cells. Cells were cultured on ampicillin agar plates, and single colonies were selected for liquid culture inoculation. Recombinant plasmids were purified with an I-Blue Mini Plasmid Kit (IBI Scientific, Peosta, IA) and digested with *Eco*RI. The presence of ~1.6 kb inserts was verified with a 0.9% agarose gel stained with ethidium bromide. Plasmid samples were submitted to the UIUC Core Sequencing Facility (Urbana, Illinois, U.S.A.) for sequencing using the primers listed in Table 1. All primers were specifically designed to amplify the *PPX2* cDNA except for M13F and M13R, which are vector primers. Reverse complements of the internal primers were also designed to sequence the antisense strand of the *PPX2* cDNAs as necessary. Following final sequence editing and quality assessments, cDNAs from one ACR plant (ACR5), one SEN plant (SEN3), and three SIR plants (SIR2, SIR4, and SIR6) were obtained. In ACR and SEN, *PPX2* was sequenced from one plant to verify their genotype (PPO inhibitor-resistant or -sensitive, respectively). SIR2A and SIR2B are cDNA sequences from the same plant (i.e., RNA sample) but were derived from separate colonies from the same *E*. *coli* transformation. DNA sequences were translated and amino acids were aligned with waterhemp PPO2 sequences from resistant (accession ABD52328.1) and sensitive (accession ABD52326.1) populations using multiple sequence alignment software (Clustal Omega, www.ebi.ac.uk/Tools/msa/clustalo/) (S1 Fig).

### Carfentrazone-ethyl and malathion interaction study with the SIR population

Plants from the SIR population were propagated using the same methods described in previous sections. Similar methods described by Ma *et al*. [11] were used for the CE and malathion treatments in this experiment. The field rate of CE (14 g ai ha^-1^) was chosen because initial greenhouse results indicated this rate did not control SIR plants, which would allow for detection of potentially increased efficacy between CE alone and CE in combination with a P450 metabolic inhibitor, malathion [11]. Four treatments included a control (adjuvants only), malathion-only, CE-only, and malathion plus CE, with five plants (each plant representing one replication) subjected to each treatment, arranged in a completely randomized design.

Foliar treatments included 0.25% (v/v) NIS to control plants or an application of 2000 g ai ha^-1^ malathion plus 0.25% (v/v) NIS to malathion-only and malathion plus CE plants. One hour after treatment, a 1% (v/v) COC plus 2.5% (v/v) AMS solution was applied to control plants or an application of 14 g ai ha^-1^ CE plus 1% (v/v) COC plus 2.5% (v/v) AMS to CE-only and malathion plus CE plants. At 2 DAT, a soil drench of 5 mM malathion solution (50 mL pot^-1^) was applied to pots where plants had previously received a foliar malathion treatment or 50 mL of deionized H_2_O was applied to control and CE-only plants. Pictures of injury were taken and plants were then harvested and dried at 10 DAT using the same methods described in previous sections. This experiment was performed independently two times with five replications per treatment, and the combined dry weights of all plants from each treatment were compared to the dry weight of control plants.

### Statistical analysis

To analyze the response of waterhemp populations to PPO-inhibiting herbicides in the greenhouse, data were transformed via a reciprocal transformation in order to achieve homogeneous variance, and the least square (LS) means were used for subsequent statistical analysis. The O’Brien test for homogeneity of variance was not significant, and the data from the two repetitions of the experiment were pooled. Data were subjected to ANOVA (SAS version 9.4) using PROC GLM. A mean separation was performed with Fisher’s Protected LSD (α = 0.1), and back-transformed data are presented in Table 2.

**Table 2:** Mean waterhemp biomass after treatment with field-use rates of PPO-inhibiting herbicides in the greenhouse.

Mean Dry Weight Percent of Nontreated Control										
Population	Carfentrazone- ethyl		Saflufenacil		Flumiclorac- pentyl		Fomesafen		Oxyfluorfen	
ACR	90.8	a	11.9	a	42.2	a	7.9	ab	14.4	a
SIR	97.9	a	10.8	ab	54.6	a	10.1	a	10.0	b
NEB	23.7	b	8.8	abc	48.8	a	9.0	ab	7.1	c
WCS	13.3	c	8.5	bc	9.9	b	7.0	b	7.3	c
SEN	18.9	bc	7.4	c	13.3	b	7.0	b	6.5	c

Back-transformed data are shown as described in Materials and Methods. Fisher’s protected LSD = 0.031 (α = 0.1) was used to detect significant differences for the transformed data. For each herbicide treatment, data that share the same letter are not significantly different.

For the CE dose-response analysis and CE plus malathion interaction study, the O’Brien test for homogeneity of variance was not significant so data from each independent experiment were pooled. These data were then subjected to ANOVA as described above. Using the dose-response curve package in R for the CE dose-response analysis, GR_50_ values and R/S ratios were estimated with nonlinear regression analysis [20]. PROC NLIN (SAS version 9.4) was used to determine values for the upper and lower asymptotes of the dose-response graph, which were 111 and 5.3 percent, respectively [21]. For the CE plus malathion interaction study, a mean separation was performed using Fisher’s Protected LSD (α = 0.05).

## Results and discussion

### Response of waterhemp populations to PPO-inhibiting herbicides in the greenhouse

Overall, saflufenacil, fomesafen and oxyfluorfen more effectively controlled each waterhemp population than flumiclorac-pentyl and CE, as the dry weight accumulation was less than 15% of their respective controls for each population (Table 2). For the flumiclorac-pentyl treatment, ACR and the two HPPD inhibitor-resistant populations accumulated significantly more dry weight than either of the sensitive populations. ACR and SIR accumulated more dry weight than the two sensitive populations when treated with CE but displayed sensitivity to saflufenacil, fomesafen, and oxyfluorfen. With the exception of the flumiclorac-pentyl treatment, NEB was not different from either sensitive population (Table 2). Due to the lack of phenotypic and quantitative evidence for PPO-inhibitor resistance, NEB was not included for subsequent studies. Based on the results of this experiment, we hypothesized that SIR was the only population in our study possessing a NTSR mechanism for PPO-inhibitor resistance (specifically CE).

ACR did not display resistance to fomesafen, oxyfluorfen, or saflufenacil as expected under our greenhouse conditions, with dry weight values ranging from 7.9% to 14.4% of nontreated controls (Table 2). However, reported GR_50_ values for fomesafen and lactofen in ACR (8 and 21 g ai ha^-1^, respectively) [17] are well below typical postemergence field-use rates for these herbicides in soybean (350 and 175 g ai ha^-1^, respectively). Other studies demonstrated that PPO inhibitor-resistant waterhemp populations are typically more resistant to the herbicide that imposed the selection pressure, which in the case of ACR is lactofen [17], and are relatively more sensitive to other subclasses having the same site of action [22–24].

### Carfentrazone-ethyl dose response

**Fig 1:**
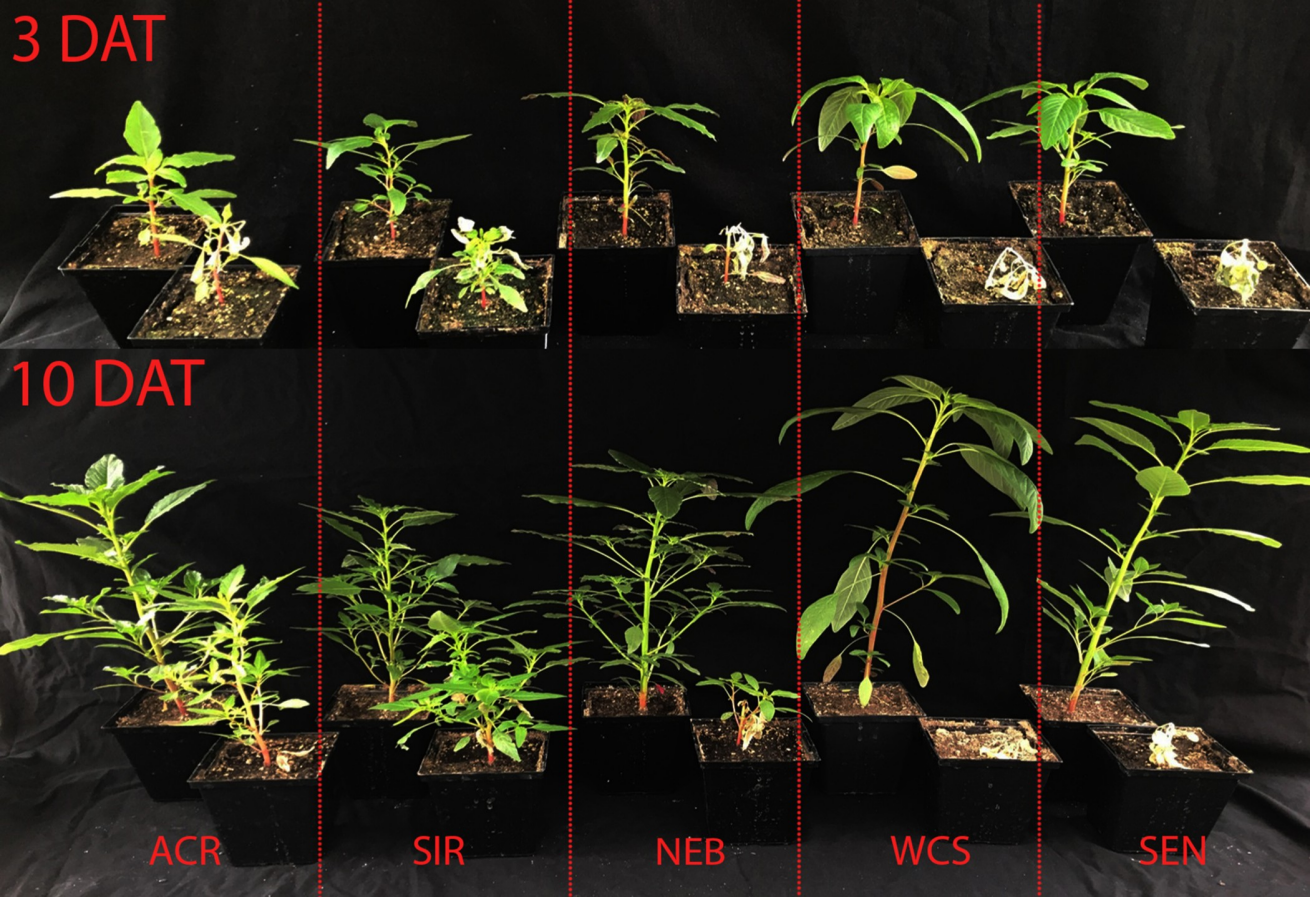
Waterhemp populations treated with a field-use rate of carfentrazone-ethyl (CE). Plants in the front row of the 3 and 10 DAT pictures were treated with 14 g ai ha^-1^ of CE (plus adjuvants) when seedlings were 9–11 cm tall. The corresponding nontreated controls for each population are positioned to the back-left of treated plants.

Based on the results of the prior experiment, ACR, SIR, and SEN were chosen to conduct a detailed CE dose-response study to quantify and compare resistance levels of SIR and ACR. CE was chosen because SIR demonstrated resistance towards CE at a field-use rate (Table 2, Fig 1). While reduced activity was observed following flumiclorac-pentyl treatment in ACR and SIR, a detailed dose-response study was not performed because this herbicide is not labeled for waterhemp control. The ACR (uniformly lactofen-resistant, possessing the ΔG210 mutation) [6, 13, 19] and SEN populations were chosen as positive and negative controls, respectively, for comparison to SIR.

**Fig 2:**
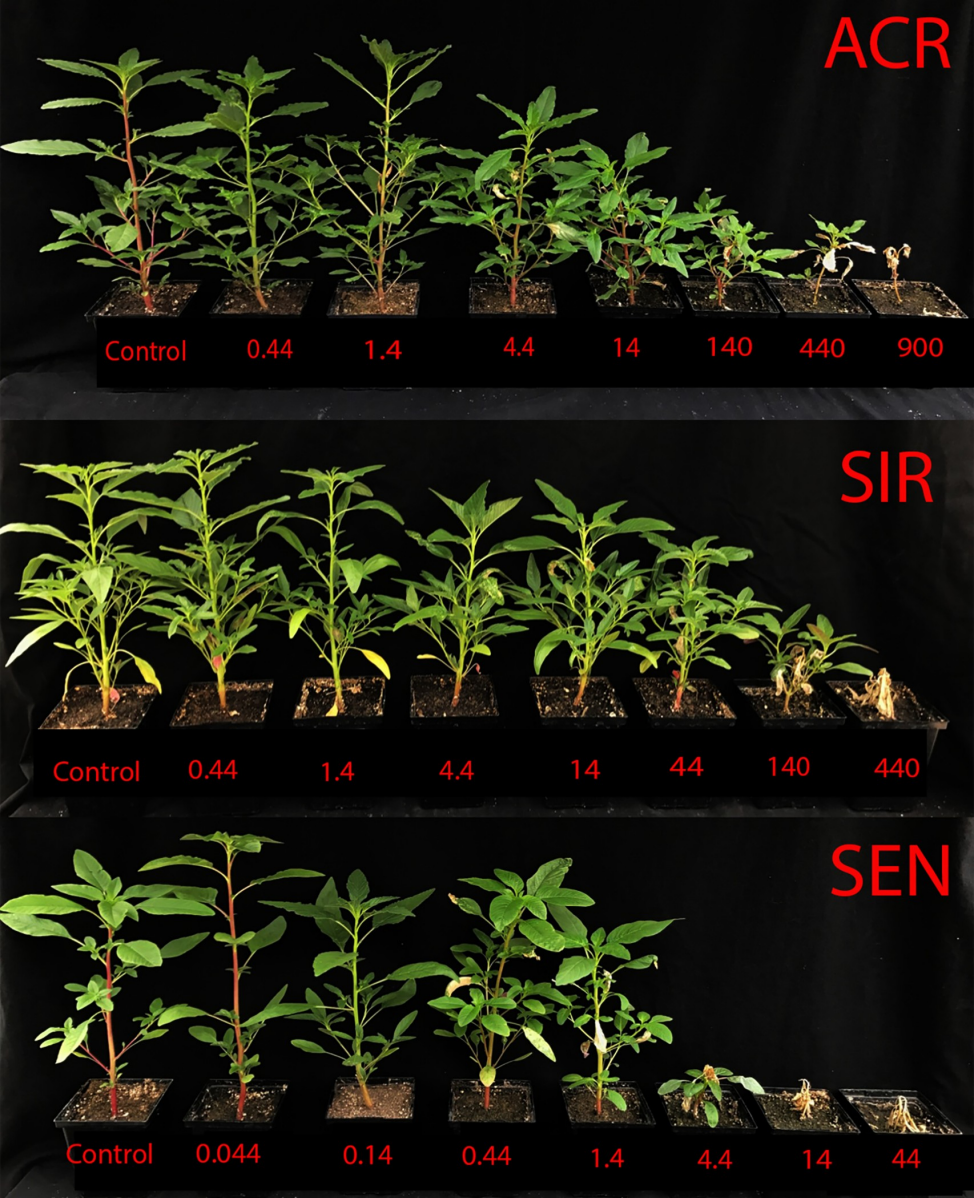
Carfentrazone-ethyl (CE) dose-response study with the ACR, SIR, and SEN populations at 10 DAT. The rates of CE (plus adjuvants) applied when seedlings were 9–11 cm tall are listed below each pot in g ai ha^-1^, while control plants were only treated with 1% (v/v) COC and 2.5% (v/v) AMS. The field-use rate for CE is 14 g ai ha^-1^.

**Fig 3:**
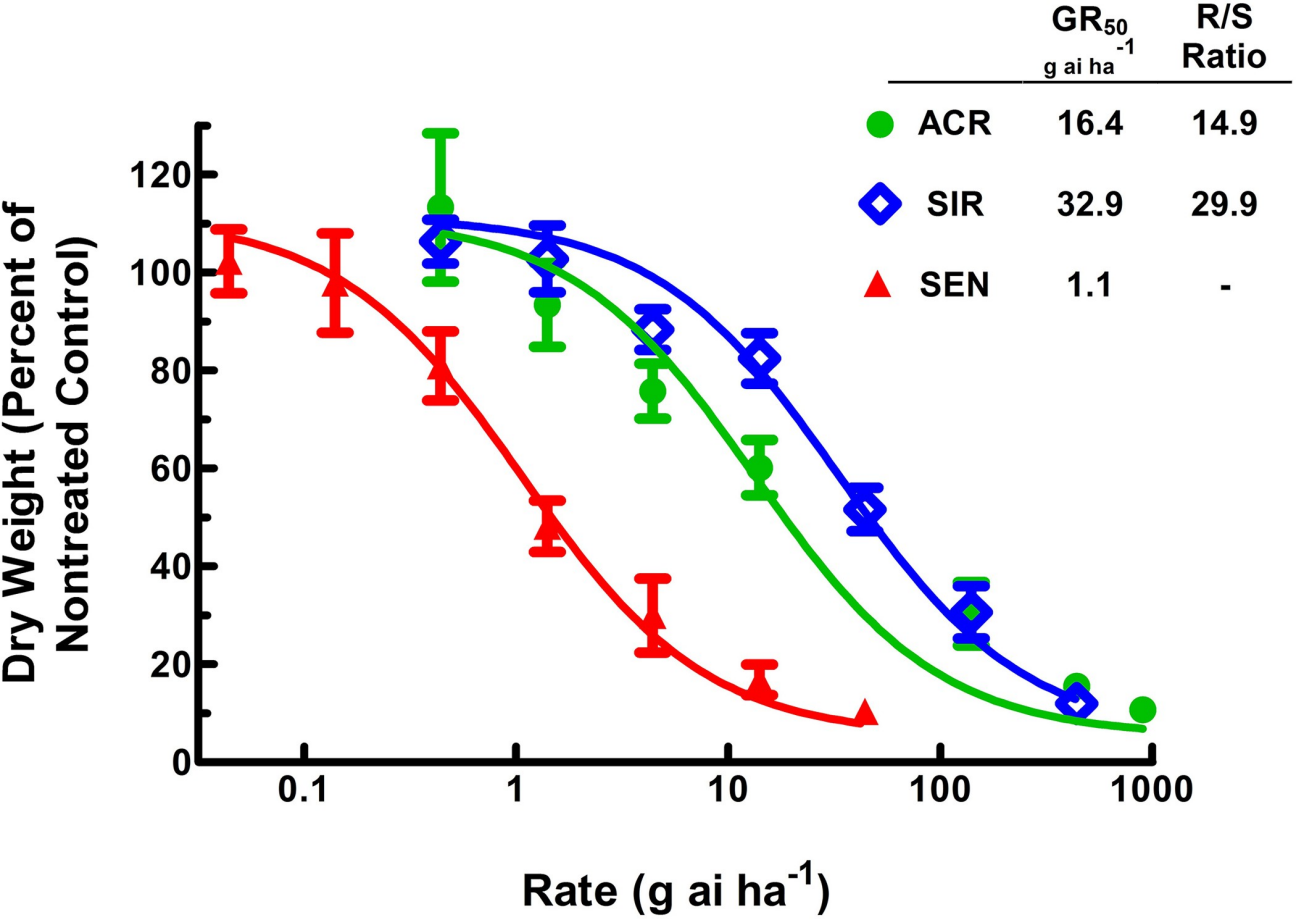
Quantitative dose-response analysis of carfentrazone-ethyl (CE) with the ACR, SIR, and SEN populations at 10 DAT. The fold-resistance ratio (R/S ratio) is the quotient of the GR_50_ from the specified resistant population (ACR or SIR) and the GR_50_ of SEN. The field-use rate for CE is 14 g ai ha^-1^, and seedlings were 9–11 cm tall at time of treatment. Vertical bars represent the standard error of the mean. Dry weights are expressed as a percentage of the nontreated control.

Phenotypic differences were observed among the three populations at 10 DAT with the various CE rates tested (Fig 2). Quantitative analysis of dry weight accumulations for each population yielded GR_50_ estimates for ACR, SIR, and SEN of 16.4, 32.9, and 1.1 g ai ha^-1^, respectively (Fig 3). When compared to SEN, ACR and SIR were approximately 15- and 30-fold resistant to CE, respectively. However, when SIR was compared to ACR, SIR was two-fold more resistant, indicating the mechanism in SIR confers a higher level of CE resistance than the PPO2 ΔG210 codon deletion in ACR.

### Sequence comparisons among deduced PPO2 proteins in waterhemp populations

When comparing deduced PPO2 amino acid sequences from SIR, ACR, and SEN with PPO inhibitor-resistant (GenBank accession ABD52328.1) and sensitive (GenBank accession ABD52326.1) waterhemp sequences, only ACR5 and ABD52328.1 possessed the ΔG210 codon deletion. However, an arginine substitution (e.g., R128G/M in Palmer amaranth [7]) was not identified in any cDNAs at nucleotides 382–384, the corresponding position in waterhemp PPO2 (S1 Fig). Several amino acid substitutions occurred in SIR sequences but none were consistent among all SIR sequences, indicating that resistance is likely not target-site mediated. SIR2A and SIR2B (cDNA sequences from the same RNA sample but derived from separate colonies from a single *E*. *coli* transformation) varied slightly in their PPO2 sequences, possibly because this plant was heterozygous at the *PPX2* locus. An alternative mechanism conferring target-site resistance could be a mutation in *PPX1*, which is a nuclear gene that only encodes the plastid isoform of PPO [4,6]. Other possible target-site resistance mechanisms include *PPX2* gene amplification or increased *PPX2* expression.

Another interesting finding was that the SEN3 PPO2 protein contained an extra glycine between glycine-251 and 252 (S1 Fig) that is due to an insertion of three nucleotides, resulting in a repeat of the codon ‘GGA’ three times. This polymorphism has been previously detected in other waterhemp populations [6] and also occurs in the cultivated grain amaranth (*A*. *hypochondriacus*) PPO2 homolog (GenBank accession ABS72165.1). This variable glycine repeat region and ΔG210 both arise from microsatellites in *PPX2*. Microsatellites are considered a mechanism for generating adaptive genetic variation [6]. Unlike the microsatellite region associated with the ΔG210 codon deletion, the extra glycine between glycine-251 and 252 does not contribute to PPO-inhibitor resistance [6], which is supported by sensitivity of the SEN population to several classes of PPO inhibitors (Table 2).

In addition to the three PPO2 sequences from SIR (S1 Fig), another cDNA named SIR6 obtained via RT-PCR revealed that variability exists in mRNAs transcribed from *PPX2*, possibly arising from a different but similar *PPX2* gene in the waterhemp genome. For example, the SIR6 *PPX2* cDNA is approximately 25-bp shorter compared to other SIR cDNA sequences. When compared to the *PPX2* cDNA sequence of *A*. *hypochondriacus* (GenBank accession EU024569.1), the SIR6 cDNA sequence is missing a portion of Exon 3 (S2A Fig) and two bases in Exon 17 (S2B Fig). In addition to these missing bases, the SIR6 *PPX2* cDNA appears to contain an insertion, which corresponds to bases 3883–3925 (portion of Intron 6) in the *A*. *hypochondriacus* genomic DNA sequence (S2A Fig). The missing and additional bases observed in the SIR6 cDNA sequence cause frameshifts that are not present in the other three sequenced *PPX2* cDNAs from SIR, indicating it may represent an alternate splice variant derived from a functional *PPX2* gene or mRNA transcribed from a pseudogene [25, 26]. In order to test these hypotheses, additional sequencing would be necessary to identify the functional *PPX2* transcript and PPO2 protein in SIR6. Additionally, it is important to note the SIR6 *PPX2* cDNA (S2 Fig) does not contain any sequence polymorphisms associated with PPO-inhibitor resistance [6–9], consistent with the other SIR cDNA sequences in our study.

CE detoxification occurs in tolerant crops via oxidative metabolism, presumably catalyzed by P450(s) [14, 15]. Metabolism-based resistance to CE, particularly through enhanced P450 expression and/or activity, thus remains a strong possibility in SIR. It is not uncommon for a single species to develop both target-site mediated and NTSR mechanisms to the same herbicide [27], as evidenced by *Lolium* and *Eleusine* populations possessing glyphosate resistance through *EPSPs* gene mutation(s) and/or reduced translocation to meristems [28, 29]. Furthermore, the multiple-resistant MCR waterhemp population displayed acetolactate synthase (ALS)-inhibitor resistance [13] through both target site and metabolism-based mechanisms [30, 31].

Although it is uncommon for metabolic resistance mechanisms to confer a greater magnitude of resistance than target-site mechanisms (e.g., refer to ALS- and photosystem II inhibitor-resistant weed species [17, 31]), NTSR mechanisms to glyphosate in grass weeds typically confer higher fold-resistance levels than mutations in *EPSPs* [29]. However, future experimentation to measure metabolism rates and detoxification pathways for CE in SIR is necessary to directly test our hypothesis of metabolism-based resistance. Preemergence applications of an aryl-triazinone or *N*-phenylphthalimide with residual activity (such as sulfentrazone or flumioxazin, respectively) would be relevant to weed management to determine if SIR is sensitive to preemergence applications, as with PPO inhibitor-resistant waterhemp populations containing the ΔG210 codon deletion [23, 32]. Although typically applied preemergence, sulfentrazone applied postemergence to SIR plants would of interest to determine if this novel resistance mechanism affects all aryl-triazinones or is in fact compound specific to CE.

### Carfentrazone-ethyl and malathion interaction study with the SIR population

**Fig 4:**
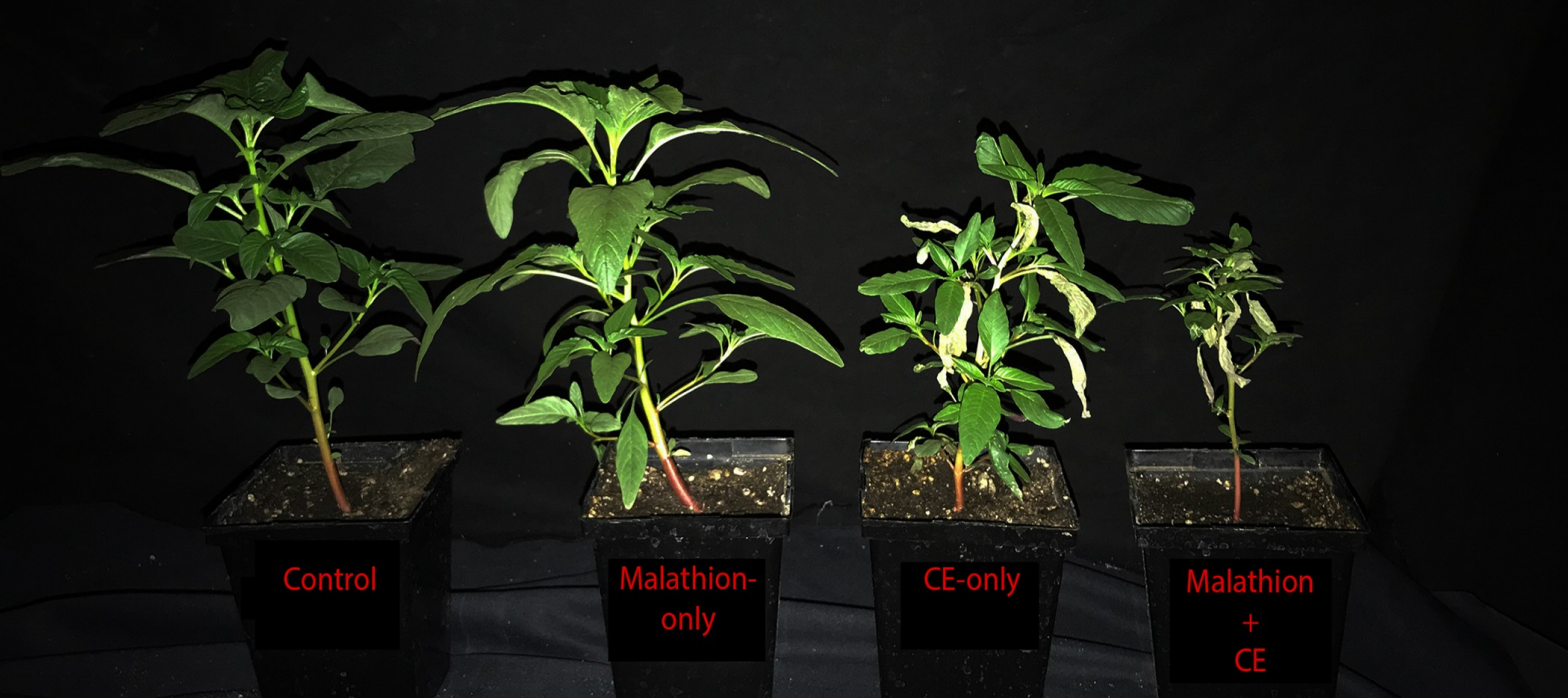
SIR plants 10 days after treatments with carfentrazone-ethyl (CE) and malathion applied alone and in combinaton. Plants at a height of 9–11 cm were treated with either adjuvants only (control), 2000 g ai ha^-1^ malathion (one hour before CE) plus a 5 mM malathion solution soil drench (50 mL pot^-1^) two days later, 14 g ai ha^-1^ CE, or malathion plus CE with the corresponding adjuvants described in Methods. Pictures of injury were taken and aboveground biomass was harvested at 10 DAT to determine dry weight accumulations per treatment. Two independent experiments were conducted with five replications each. Combined means for each treatment (expressed as a percentage of the adjuvant-only control) were 90.8% for malathion-only, 36.5% for CE-only, and 15.2% for the combination of malathion plus CE. A Fisher’s protected LSD value of 13.0 (α = 0.05) was used to determine significant differences among treatments.

Plants treated with both CE and malathion exhibited more foliar injury and accumulated less dry weight (15.2% of the adjuvant-only control) than plants treated with CE-only (36.5% of the adjuvant-only control), and both treatments were significantly less than plants treated with malathion-only (90.8% of the adjuvant-only control; Fig 4). The results indicated that CE is more phytotoxic to SIR plants in the presence of malathion, consistent with the hypothesis that CE resistance in SIR is due to enhanced oxidative metabolism via P450s [33]. However, the actual number of P450 enzymes and extent of activity inhibition by malathion in plants is currently unknown. Additional research examining the metabolism of CE in SIR foliar tissues, with and without metabolic inhibitors [30], is needed to further investigate this hypothesis and to confirm the involvement of P450 activity with *in vitro* microsomal assays using CE and carfentrazone acid as substrates [14, 33].

## Conclusions

These experiments demonstrated a novel resistance mechanism that affects CE but not other PPO-inhibiting herbicides examined so far in the SIR population. The precise mechanism has yet to be determined, but it is not due to known PPO2 mutations in *Amaranthus* [6–9]. Target-site mediated resistance to CE remains possible, such as *PPX2* copy number variation, increased *PPX2* expression, or a mutation in the *PPX1* gene [4]. The results of the CE treatment in combination with malathion suggest that SIR possesses enhanced metabolism of CE via P450s. An NTSR mechanism such as this could potentially affect CE but not DPEs or saflufenacil in SIR. In support of this theory, metabolic resistance to atrazine in the MCR population [11] did not confer cross-resistance to metribuzin [34], an asymmetrical triazine.

New mechanisms of resistance may affect commercial products currently being developed. Not only is there a continuing desire to create genetically modified crops resistant to PPO-inhibiting herbicides, which has already been achieved with engineered maize and rice (*Oryza sativa*) varieties [35], but a novel benzoxazinone PPO inhibitor (trifludimoxazin) is currently under development [36]. It is possible that SIR and certain *Amaranthus* populations [9] will not be controlled with new PPO-inhibiting herbicides currently in development through these yet-to-be determined, novel resistance mechanism(s). Current PCR-based diagnostic assays designed to detect the known sequence polymorphisms in *PPX2* [8, 9] would not detect CE resistance in waterhemp or Palmer amaranth populations with a NTSR mechanisms, resulting in false negatives. Furthermore, if CE resistance in SIR were conferred by a NTSR mechanism, the possibility of SIR plants crossing with waterhemp plants containing any of the currently known target-site mutations [4–8] would result in progeny possessing both NTSR and target-site resistance mechanisms, and potentially a greater level of resistance to CE or other PPO-inhibiting herbicides. Nevertheless, the discovery and development of new active ingredients and PPO inhibitor-resistant crops will likely lead to an increase in the use of old and new PPO-inhibiting herbicides, which will increase the selection pressure for multiple resistance in weeds and could lead to an increased occurrence of both target-site and NTSR mechanisms.

## Supporting information

S1 Fig Deduced amino acid sequences of waterhemp PPO2 proteins. One partial ACR cDNA, one partial SEN cDNA, and three partial SIR cDNAs were aligned with corresponding PPO2 sequences from a resistant (accession ABD52328.1) and sensitive (accession ABD52326.1) waterhemp population from GenBank. Positions for arginine-128 [8], glycine-210 [6], and the polymorphic glycine in SEN3 that does not confer PPO-inhibitor resistance are highlighted in yellow, green, and blue, respectively. For sequence comparisions, an asterisk indicates positions that have a single, fully conserved residue; a colon indicates conservation among residues possessing strongly similar properties; and a period indicates conservation among residues possessing weakly similar properties. Amino acid numbering is based on the ABD52326.1 protein sequence from GenBank. Sequences were aligned using the Clustal Omega software.
(TIFF)

S2 Fig Portions of the SIR6 *PPX2* cDNA aligned with the corresponding regions of the *A. hypochondriacus PPX2* cDNA (GenBank accession EU024569.1). Only portions of the complete alignment showing potential splicing deviations in the putative pseudogene-derived SIR6 *PPX2* transcript (A) and sites with known mutations in *Amaranthus PPX2* (B) are presented. (A) Nucleotides highlighted in yellow indicate the portion of Exon 3 missing in the SIR6 cDNA. Nucleotides highlighted in red indicate the locations of target-site mutations that confer PPO-inhibitor resistance in *Amaranthus* (point mutations G114E, R128G/M, S149I or a codon deletion ΔG210), which are not present in the SIR6 cDNA. Note the single base change in SIR6 at position 563 (relative to EU024569.1) does not alter the encoded protein. Nucleotides highlighted in blue indicate the portion of Intron 6 (from GenBank accession EU024569.1 genomic DNA) that is present in the SIR6 *PPX2* cDNA. (B) Nucleotides highlighted in red indicate the location of a known target-site mutation that confers PPO-inhibitor resistance in *Amaranthus palmeri* (G399A), which is not present in the SIR6 cDNA. Nucleotides highlighted in green indicate the two missing bases from Exon 17 in the SIR6 cDNA. Nucleotide numbering in panels A and B is based on the EU024569.1 cDNA.
(TIFF)
