# Supplementary figures and images for: Carfentrazone-ethyl resistance in an Amaranthus tuberculatus population is not mediated by amino acid alterations in the PPO2 protein
Source: PLoS One. 2019 Apr 15;14(4):e0215431. doi: 10.1371/journal.pone.0215431 (PMC6464220; doi:10.1371/journal.pone.0215431)

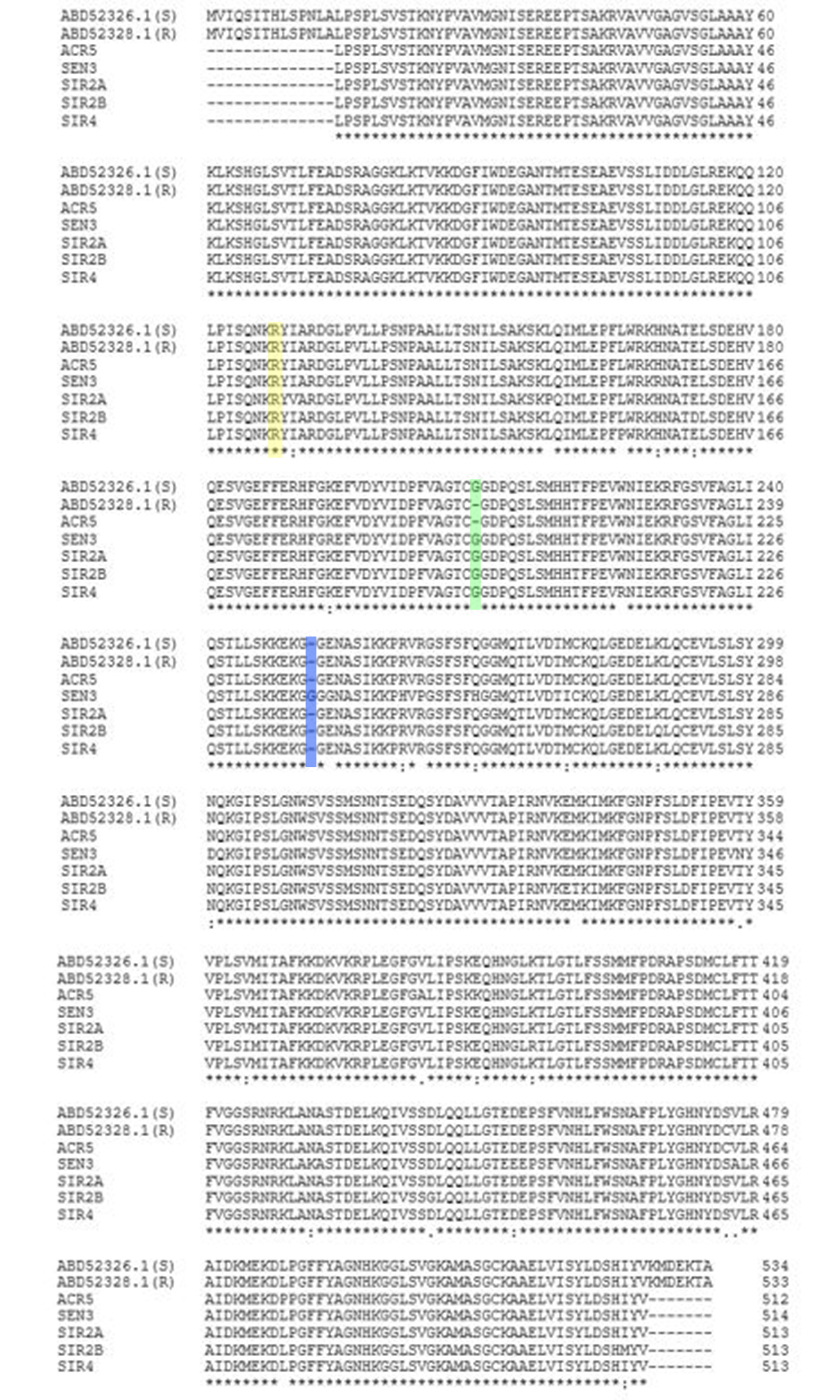

Supplement: S1 Fig — One partial ACR cDNA, one partial SEN cDNA, and three partial SIR cDNAs were aligned with corresponding PPO2 sequences from a resistant (accession ABD52328.1) and sensitive (accession ABD52326.1) waterhemp population from GenBank. Positions for arginine-128 [8], glycine-210 [6], and the polymorphic glycine in SEN3 that does not confer PPO-inhibitor resistance are highlighted in yellow, green, and blue, respectively. For sequence comparisions, an asterisk indicates positions that have a single, fully conserved residue; a colon indicates conservation among residues possessing strongly similar properties; and a period indicates conservation among residues possessing weakly similar properties. Amino acid numbering is based on the ABD52326.1 protein sequence from GenBank. Sequences were aligned using the Clustal Omega software. (TIFF) [file pone.0215431.s001.tiff]

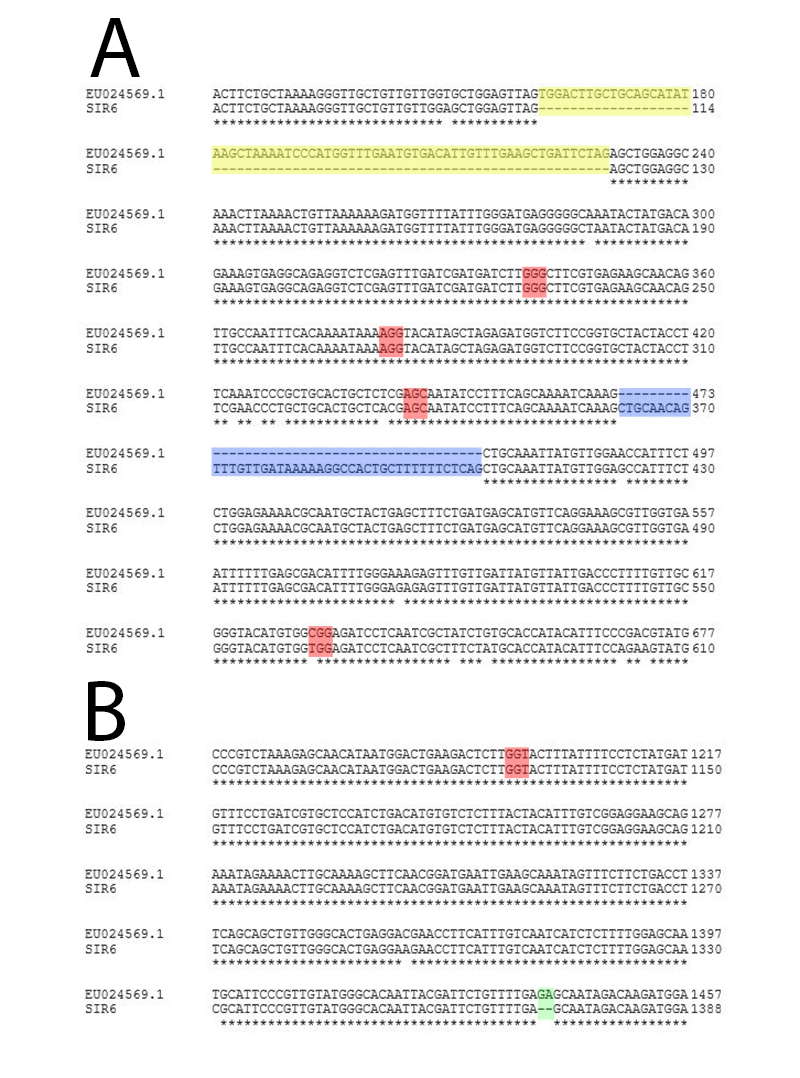

Supplement: S2 Fig — Only portions of the complete alignment showing potential splicing deviations in the putative pseudogene-derived SIR6 PPX2 transcript (A) and sites with known mutations in Amaranthus PPX2 (B) are presented. (A) Nucleotides highlighted in yellow indicate the portion of Exon 3 missing in the SIR6 cDNA. Nucleotides highlighted in red indicate the locations of target-site mutations that confer PPO-inhibitor resistance in Amaranthus (point mutations G114E, R128G/M, S149I or a codon deletion ΔG210), which are not present in the SIR6 cDNA. Note the single base change in SIR6 at position 563 (relative to EU024569.1) does not alter the encoded protein. Nucleotides highlighted in blue indicate the portion of Intron 6 (from GenBank accession EU024569.1 genomic DNA) that is present in the SIR6 PPX2 cDNA. (B) Nucleotides highlighted in red indicate the location of a known target-site mutation that confers PPO-inhibitor resistance in Amaranthus palmeri (G399A), which is not present in the SIR6 cDNA. Nucleotides highlighted in green indicate the two missing bases from Exon 17 in the SIR6 cDNA. Nucleotide numbering in panels A and B is based on the EU024569.1 cDNA. (TIFF) [file pone.0215431.s002.tiff]
